# 

**Published:** 1891-09

**Authors:** 


### CONTENTS.

### COMMUNICATIONS.

An Ideal College Curriculum.......... 421
Chloralamid in Surgery.......... 435

### PROCEEDINGS.

Thirty-First Annual Meeting of the American Dental Association... 438
Tennessee's Dental Law.......... 446

### SELECTIONS.

The Creosote Treatment of Tuberculosis.......... 449
How to Clean Old Slides and Utilize Spoiled Mounts.......... 451
Bromide of Ethyl in Dental Practice.......... 452
Statistics of Anæsthetics.......... 453
Hipocrates on Injuries to the Skull—Their Surgical Treatment...... 454
The Transplantation of Teeth.......... 455
Transmissibility of Syphilis.......... 456
Medical Properties of Vegetables.......... 457
Teeth in Diabetes.......... 458
Drinking Ether.......... 458
A simple Method of Curing Obesity.......... 459
Effects of Strychnine on the Stomach.......... 460
Physiolgy in the Schools.......... 460
Lukewarm Baths to Restore the Waning Power of Age to Youthful Vigor.......... 461
How to Extinguish Fire.......... 461
The Monkey Solves the Problem.......... 462
Pathology of Grief.......... 462

### EDITORIAL.

The American Dental Association.......... 464
New Dental Colleges.......... 466
The Indiana State Dental Association.......... 467
Office Plans.......... 469

